# Rifting of the oceanic Azores Plateau with episodic volcanic activity

**DOI:** 10.1038/s41598-020-76691-1

**Published:** 2020-11-12

**Authors:** B. Storch, K. M. Haase, R. H. W. Romer, C. Beier, A. A. P. Koppers

**Affiliations:** 1 GeoZentrum Nordbayern, Friedrich-Alexander-Universität Erlangen-Nürnberg (FAU), Schlossgarten 5, 91054 Erlangen, Germany; 2 Department of Geosciences and Geography, Research Programme of Geology and Geophysics (GeoHel), University of Helsinki, PO Box 64, 00014 Helsinki, Finland; 3 College of Earth, Ocean and Atmospheric Sciences, Oregon State University, 104 CEOAS Admin Bldg, Corvallis, OR 97331-5503 USA

**Keywords:** Geochemistry, Geodynamics, Geology, Petrology

## Abstract

Extension of the Azores Plateau along the Terceira Rift exposes a lava sequence on the steep northern flank of the Hirondelle Basin. Unlike typical tholeiitic basalts of oceanic plateaus, the 1.2 km vertical submarine stratigraphic profile reveals two successive compositionally distinct basanitic to alkali basaltic eruptive units. The lower unit is volumetrically more extensive with ~ 1060 m of the crustal profile forming between ~ 2.02 and ~ 1.66 Ma, followed by a second unit erupting the uppermost ~ 30 m of lavas in ~ 100 kyrs. The age of ~ 1.56 Ma of the youngest in-situ sample at the top of the profile implies that the 35 km-wide Hirondelle Basin opened after this time along normal faults. This rifting phase was followed by alkaline volcanism at D. João de Castro seamount in the basin center indicating episodic volcanic activity along the Terceira Rift. The mantle source compositions of the two lava units change towards less radiogenic Nd, Hf, and Pb isotope ratios. A change to less SiO_2_-undersaturated magmas may indicate increasing degrees of partial melting beneath D. João de Castro seamount, possibly caused by lithospheric thinning within the past 1.5 million years. Our results suggest that rifting of oceanic lithosphere alternates between magmatically and tectonically dominated phases.

## Introduction

Oceanic plateaus with a crustal thickness to 30 km cover large areas in the oceans and these bathymetric swells affect oceanic currents and marine life^1,2^. Most oceanic plateaus have complex magmatic histories with several volcanic phases erupting tholeiitic to alkaline basaltic lavas over time scales of tens of millions of years^3–6^. For example, drilling of Pacific oceanic plateaus revealed that the Ontong-Java Plateau apparently formed between 121 and 37 Ma by four volcanic episodes, whereas the Shatsky Plateau erupted continuously between 144 and 129 Ma^4^. The main magmatic episode forming oceanic plateaus is believed to reflect the initial arrival of a deep mantle plume head e.g.^5^, but the overall sequence that follow the mantle and volcanic processes in oceanic plateau remains poorly understood^4^. Stratigraphic sampling of continental flood basalt lava flows yields important insight into petrogenetic processes^7^, but similar studies at oceanic plateaus have been limited by the depths of drill cores that typically sampled the uppermost few hundred meters^4,6^. Oceanic plateaus frequently show evidence of rifting phases like, for example, the Manihiki and Kerguelen Plateaus^8,9^. The Azores Plateau formed 10 to 4 million years ago^10^ and is rifted by the NW–SE striking ultraslow Terceira Rift^11–13^. Seismic work suggested an opening of the Terceira Rift ~ since 25–20 Ma ago^14^, whereas tectonic studies suggested rifting initiation 1to 2 Ma ago^15,16^. Deep submarine rift basins of the Terceira Rift are results of the extension and expose the earlier volcanic stages along the 1 to 2 km high escarpments of the rift flanks. Volcanic edifices with ages < 1.5 Ma formed within the Terceira Rift^17–20^ causing a morphology that resembles the magmatic and amagmatic segments at ultraslow-spreading centers such as the Southwest Indian Ridge and the Gakkel Ridge in the Arctic Ocean^21,22^. Large volcanic structures imply short-lived melt focusing at the magmatic segments, whereas mantle peridotite occurs in deep sediment-covered amagmatic ridge segments^21,23^. Magmatic segments with average lengths of 25 to 60 km are also typical for the continental Main Ethiopian rift system with a significantly thicker lithosphere than slow-spreading mid-ocean ridges^24^. The magmatic intrusions reduce the strength of the lithosphere and thus play an important role in the rifting process^25^.

Here, we present geochronological and geochemical data on the upper 1.2 km of the Azores Plateau crust that give evidence for episodic volcanic activity at the Terceira Rift. The new data show that the Terceira Rift opened after 1.56 Ma with tectonic extension followed by volcanism in the rift basin. The basanitic to alkali basaltic magmas form by low degree (< 5%) partial melting beneath thick lithosphere and the increasing SiO_2_ contents of primitive melts with time probably reflect rifting-induced progressive lithospheric thinning and increasing degrees of melting at shallower depths.

### Geological setting

**Figure 1:**
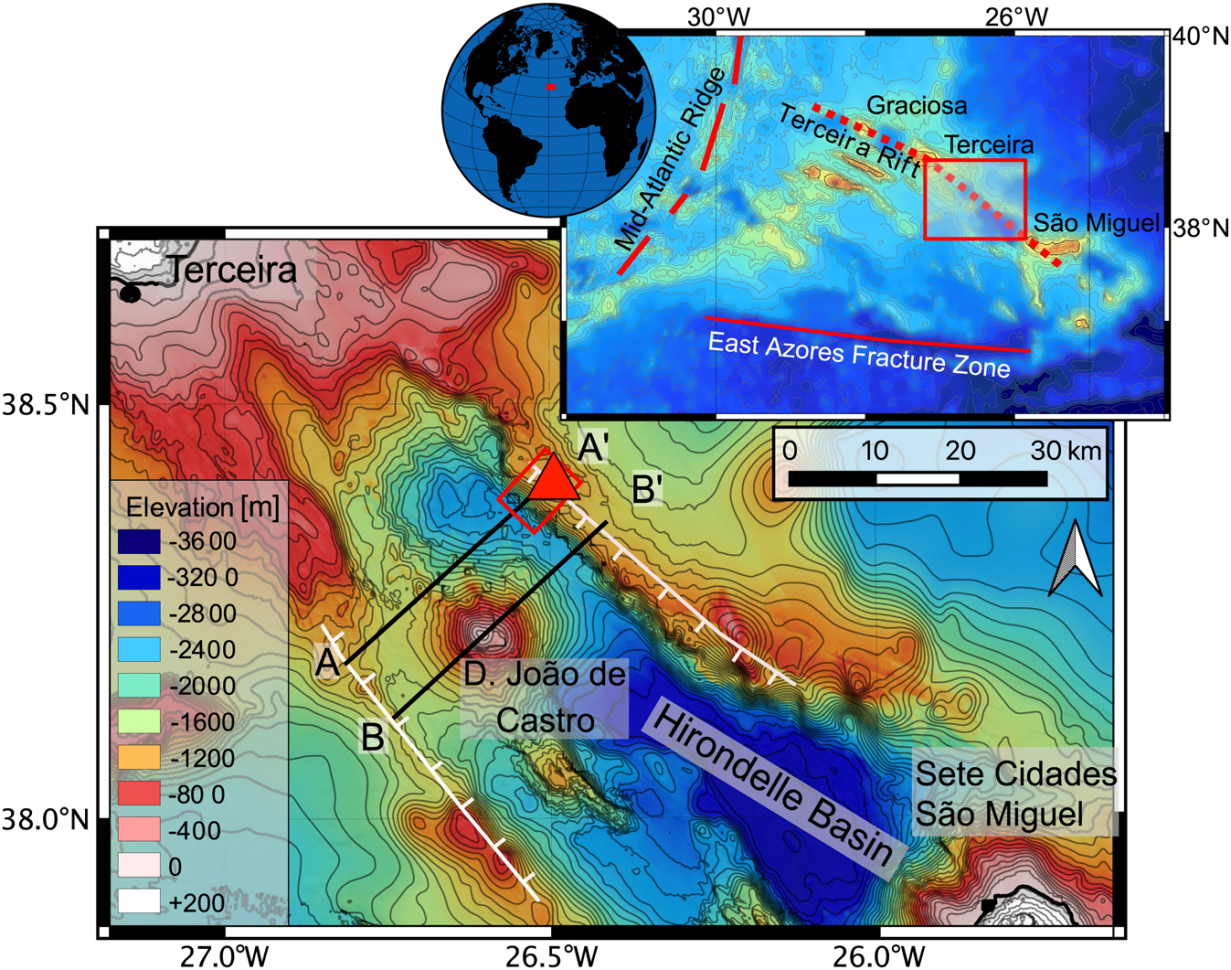
Bathymetric maps of the Azores Plateau in the North Atlantic with the tectonic structures of the Terceira Rift, the Mid-Atlantic Ridge, and the East Azores Fracture Zone shown in red in the smaller map. The small red square marks the sampling area, that is shown in more detail in the enlarged bathymetric map. The large map shows the bathymetry of the Hirondelle Basin between the islands of Terceira and Sete Cidades volcano on São Miguel. Bathymetric grids are combined ship-based multibeam maps from RV *Meteor* cruises M113, M128 and^39^. The red triangle marks the location of the stratigraphic profile sampled during M128. The northern graben shoulder, as well as the southern flank appear to represent normal faults shown as white lines that caused basin opening. The black lines A-A’ and B-B’ indicate the profiles shown in Fig. 7. Map created using QGIS 3.4 Madeira (2018). QGIS Geographic Information System. Open Source Geospatial Foundation Project. https://qgis.org.

The Azores Plateau covers an area of ~ 4 × 10^5^ km^2^ ^26^ with a minimum crustal thickness of ~ 16 km^27,28^, thus representing a slightly smaller oceanic plateau than Shatsky Rise in the NW Pacific with an area of 5.33 × 10^5^ km^2^ ^29^. Large portions of the Azores Plateau probably formed by enhanced melt production close to the Mid-Atlantic Ridge (MAR) between 10 and 4 Ma ago, possibly with the abundant eruption of tholeiitic basalts from large melt volumes in the head of a deep mantle plume^10,30^. In contrast, most of the Azores islands are younger than 1.5 million years and erupt alkaline lavas^17–20^. The abundant volcanism may be caused by a small thermal mantle anomaly^31,32^, or by decompression melting of a volatile-enriched mantle^33,34^. The anomalously thick oceanic crust of the eastern Azores Plateau is bounded by the roughly N-S striking MAR in the west (Fig. 1). Extension within the Azores Plateau occurs along several NW–SE and WNW-ESE striking fault zones with the Terceira Rift being the most pronounced^11,12^. Several authors suggested the formation of new oceanic lithosphere along the Terceira Rift but no systematic magnetic anomaly pattern parallel to the Terceira Rift is observed^11,13,35^. The extension may have occurred in two phases with the first by normal faulting of existing crust of the entire Azores plateau, and the second very recent phase with magmatic intrusions along the Terceira Rift^35^. Seismic studies reveal an extended crust with numerous normal faults and suggest a NE directed migration of the rifting in the SE part of the Terceira Rift^14^. The oblique ultraslow extension of the Terceira Rift opened the Hirondelle Basin with later formation of the volcanic islands of Terceira and São Miguel^15,36^, and the large D. João de Castro seamount that occurs in the northwestern portion of the basin (Fig. 1). D. João de Castro seamount is an active volcano with reported eruptive activity in 1720 and active shallow hydrothermal venting^37^. The Hirondelle Basin is less than 35 km wide and extends ~ 100 km from SE to NW and is bounded by rift flanks rising from ~ 2500 to 1300 m below sea level (mbsl, Fig. 1). The northern rift flank is steeper than the southern flank probably reflecting the existence of several faulted blocks in the south (Fig. 1). The Hirondelle Basin is seismically active implying ongoing tectonic extension in this area^38^.

## Methods

### Major and trace elements

Most of the samples from ROV-Dives 738ROV and 789ROV (Supplementary Material I) are plagioclase ± clinopyroxene phyric moderately to highly vesicular volcanic rocks but some samples are aphyric. One volcanic glass sample was separated (IEAZO1047; 789ROV-01) and analyzed using the JEOL JXA-8200 superprobe electron microprobe at the GeoZentrum Nordbayern in Erlangen, Germany, using methods described previously^40^. Weathered surfaces and vesicle fillings were removed from the whole-rock samples prior to sample preparation. The samples were then washed, coarse crushed and powdered in an agate grinder. We carried out analyses of major and trace element concentrations at the GeoZentrum Nordbayern in Erlangen, Germany, following the procedures outlined previously^41^. The international rock standards BHVO-2, BE-N, BR and GA were repeatedly measured with the samples. The major and trace element and isotope analysis procedure is described in detail in the Supplementary Material II.

### Isotope analysis

For Sr–Nd–Pb isotope ratio analysis, about 0.10 to 0.12 g dried whole rock powder was leached passed though the separation procedures outlined previously. All used acids were Teflon distilled (those for Pb were double-distilled), and typical procedural blanks for Pb, Sr and Nd were 30 pg, 200 pg and 80 pg, respectively. Lead isotopes were measured by double spike analysis using a Thermo Scientific Neptune Plus High Resolution Multicollector ICP-MS (MC-ICP-MS) at the GeoZentrum Nordbayern in Erlangen, Germany. Repeated measurements of the NBS981 Pb isotope standard measured as an unknown over the course of this study gave ^206^Pb/^204^Pb, ^207^Pb/^204^Pb, ^208^Pb/^204^Pb ratios of 16.9391 ± 0.0018, 15.4965 ± 0.0019 and 36.7149 ± 0.0036, respectively, compared to published values of 16.9379 ± 30, 15.4932 ± 26, and 36.7013 ± 76^42^. The Sr and Nd isotope ratios were determined in static mode with a Thermo Scientific Triton Series Multicollector Thermal Ionization Mass Spectrometer (TIMS) at the GeoZentrum Nordbayern in Erlangen, Germany. Repeated analyses of the NBS987 Sr standard yielded an average value of 0.710259, and the in-house ‘Erlangen Nd’ standard solution gave ^143^Nd/^144^Nd of 0.511840, equivalent to a value of 0.511850 for the La Jolla standard.

Hafnium was separated using a modified version of published methods^43,44^. Titanium (using an oxidation mixture) and Zr were separated from the Hf fractions through further steps on Ln-Spec columns. The isotopes were measured with a Thermo Scientific Neptune Plus High Resolution MC-ICP-MS, at the GeoZentrum Nordbayern in Erlangen, Germany. We measured the AMES Grenoble standard yielding a ^176^Hf/^177^Hf 0.282171 ± 3 (n = 6) compared to a published value of 0.282169 ± 22^42^. All measured standard values and the Hirondelle Basin dataset are listed in the Supplementary Material III.

### ^40^Ar/^39^Ar ages

All ^40^Ar/^39^Ar age determinations (groundmass and plagioclase phenocrysts, see Table 1) for the Hirondelle Basin samples were carried out at the Oregon State University (OSU) Argon Geochronology Laboratory, USA (described in detail in Supplementary Material IV). The separated grain size fraction between 150 and 300 μm was washed (ultrapure water), dried at 55 °C and plagioclase phenocrysts were separated by hand-picking from groundmass material. The density fractions were acid-leached with 1 M HCl, then 6 M HCl, 1 M HNO_3_, 3 M HNO_3_ and ultra-pure deionized water (all for about 60 min) in an ultrasonic bath heated to ~ 50 °C. The plagioclase phenocrysts were leached using 5% HF for 5–15 min. The leached samples were irradiated for 6 h in the TRIGA nuclear reactor at OSU, together with the FCT sanidine flux monitor^45^. The individual J-values for each sample were calculated by parabolic extrapolation of the measured flux gradient against irradiation height and typically give 0.1–0.2% uncertainties (1σ). The ^40^Ar/^39^Ar incremental heating age was determined with two multicollector ARGUS-VI mass spectrometers. After loading the irradiated samples into Cu-planchettes in an ultra-high vacuum sample chamber, they were incrementally heated by scanning a defocussed 25 W CO_2_ laser beam in preset patterns across the sample, in order to release the Ar evenly. Each pass involved incremental heating of 15–20 mg of separated groundmass material or plagioclase phenocrysts. The sample material was ‘pre-cleaned’ for 60 s, while released gasses were pumped away directly at two low (0.5%, 1.8%) laser power settings to remove any loosely-held atmospheric Ar adsorbed onto grain surfaces. After heating, the reactive gases were cleaned out using a SAES Zr-al ST101 getter operated at 400 °C and two SAES Fe-V-Zr ST172 getters operated at 200 °C and room temperature, respectively. Samples were held in the extraction line for a total time of 6 min. Blank intensities were measured every 3 incremental heating steps for groundmass and glass, and every 2 steps for plagioclase phenocrysts. For calculating the ages, the corrected decay constant of Steiger and Jäger^46^ was used: 5.530 ± 0.097 × 10^–10^ yr^-1^ (2σ) as reported by Min, et al.^47^. Incremental heating plateau ages and isochron ages were calculated as weighted means with 1/σ^2^ as weighting factor^48^ and as YORK2 least-square fits with correlated errors^49^ using the ArArCALC v2.7.0 software Koppers^50^ available from the https://earthref.org/ArArCALC/ website. The samples were initially interpreted using the inverse isochron because such ages do not assume a ^40^Ar/^36^Ar composition for trapped Ar. Inverse isochron ages are calculated for samples with five or more data points using steps that deviate by less than 3σ from the ^39^Ar/^40^Ar and ^36^Ar/^40^Ar weighted means with a uniform distribution^51^. In addition, the isochron ages are considered robust if (1) the total released ^39^Ar (k) ≥ 50%. (2) The isochron has a spreading factor > 5% (S-factor^51^), MSWD < 1 + 2 (2/ƒ)^1/2^ ^52^, where f = n-2 and n is number of steps in the isochron, and (3) the ^40^Ar/^36^Ar intercept is within error or greater than 295.5 ± 0.7 1σ. If experiments had no resolvable isochron but yielded highly radiogenic Ar, the initial trapped ^40^Ar/^36^Ar was assumed to equal 295.5^53^, and a plateau model age was calculated.

**Table 1:** Summary of ^40^Ar/^39^Ar data.

IGSN	Sample	Cruise	Separated phase	Rock type	Plateau age (ky)	± 2σ	MSWD	^39^Ar (%)	*n* steps	^40^Ar/^36^Ar intercept ± 2σ	Inverse isochron age (ky)	± 2σ	MSWD	K/Ca	± 2σ
IEAZO0903	738-ROV-02	M128	Groundmass	Lava	**2020**	**± 10** **± 0.5%**	0.79	100	31	286.95 ± 3.89	2040	± 20 ± 0.98%	0.98	0.227	± 0.000
IEAZO1054	789-ROV-08	M128	Groundmass	Lava	**1957.9**	**± 7.7** **± 0.39%**	1.3	36	6	253.31 ± 93.54	1986.9	± 60.6 ± 3.05%	1.42	0.230	± 0.000
			Plagioclase	Lava	1815.4	± 82.6 ± 4.55%	0.93	87	17	305.01 ± 71.71	1777.2	± 251.6 ± 14.16%	0.99	0.006	± 0.000
IEAZO1064	789-ROV-17	M128	Groundmass	Lava	**1656.7**	**± 4.1** **± 0.25%**	0.43	36	6	287.57 ± 52.45	1662.8	± 40.1 ± 2.41%	0.53	0.473	± 0.001
			Plagioclase	Lava	1993.1	± 50.4 ± 2.53%	1.88	83	18	284.26 ± 24.33	1975.2	± 99.8 ± 5.05%	2.94	0.012	± 0.000
IEAZO1065	789-ROV-18	M128	Groundmass	Lava	**1558.8**	**± 4.5** **± 0.29%**	1.42	49	9	296.76 ± 6.76	1556.5	± 13 ± 0.84%	1.71	0.372	± 0.001

Preferred ages are marked in bold. Full data tables are available in the Supplementary Materials III and IV.

## Results

### Sampling and age determinations of lavas from crustal profile

**Figure 2:**
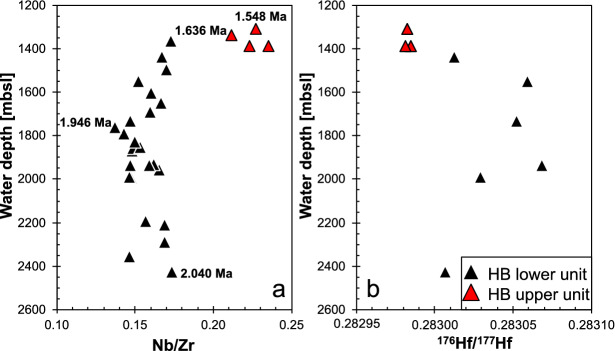
The variation of (**a**) Nb/Zr and (**b**) ^176^Hf/^177^Hf ratios versus water depth [meters below sea level. mbsl] of the samples recovered at the northern graben shoulder of the Hirondelle Basin. Samples of the lower unit are depicted as black symbols and of the upper unit in red. Note that the error bars in (**b**) are smaller than the symbols and therefore not shown in this graph. The bold numbers indicate the Ar–Ar groundmass plateau ages of selected samples.

The samples from the northern Hirondelle Basin wall (Fig. 1) were recovered by the Remotely Operated Vehicle (ROV) ‘Quest 4000’ (MARUM Bremen), during research cruise M128 in 2016 with the German RV *Meteor*. We stratigraphically sampled a ~ 1.2 km vertical profile of the northern flank of the Hirondelle Basin between 2510 and 1308 mbsl (Fig. 2). All samples were obtained from submarine pillow lava flows (Supplementary Material I) and thus represent eruptive units rather than intrusive rocks. Whereas the lower part of the profile consists only of lavas and dikes, volcaniclastic rocks and pelagic sediments become more abundant shallower than 1690 mbsl depth where they alternate with pillow lavas.

Four samples were selected for ^40^Ar/^39^Ar age dating (on groundmass and plagioclase phenocrysts) at Oregon State University, USA (Table 1). The lowermost sample IEAZO0903 (2427 mbsl) has a groundmass plateau age of 2.020 ± 0.010 Ma (Fig. 2). Sample IEAZO1054 (1760 mbsl) from the central part of the profile reveals a groundmass plateau age of 1.958 ± 0.008 Ma. The uppermost samples IEAZO1064 (1367 mbsl) and IEAZO1065 (1338 mbsl) show groundmass plateau ages of 1.657 ± 0.004 Ma and 1.558 ± 0.005 Ma, respectively. The groundmass ages are interpreted as eruption ages, yet from the inverse isochron ^40^Ar/^36^Ar intercept calculations, the samples do show evidence for (minor amounts of) excess Ar, which has been corrected accordingly^54^. The four samples cover ~ 1.2 km of vertical crustal profile representing a time interval of ~ 500 kyrs. The lower ~ 1060 m indicate formation within ~ 400 kyrs, whereas the uppermost ~ 30 m of the profile have an age difference of ~ 90 kyrs (based on the groundmass plateau ages).

### Geochemical variation within the profile

**Figure 3:**
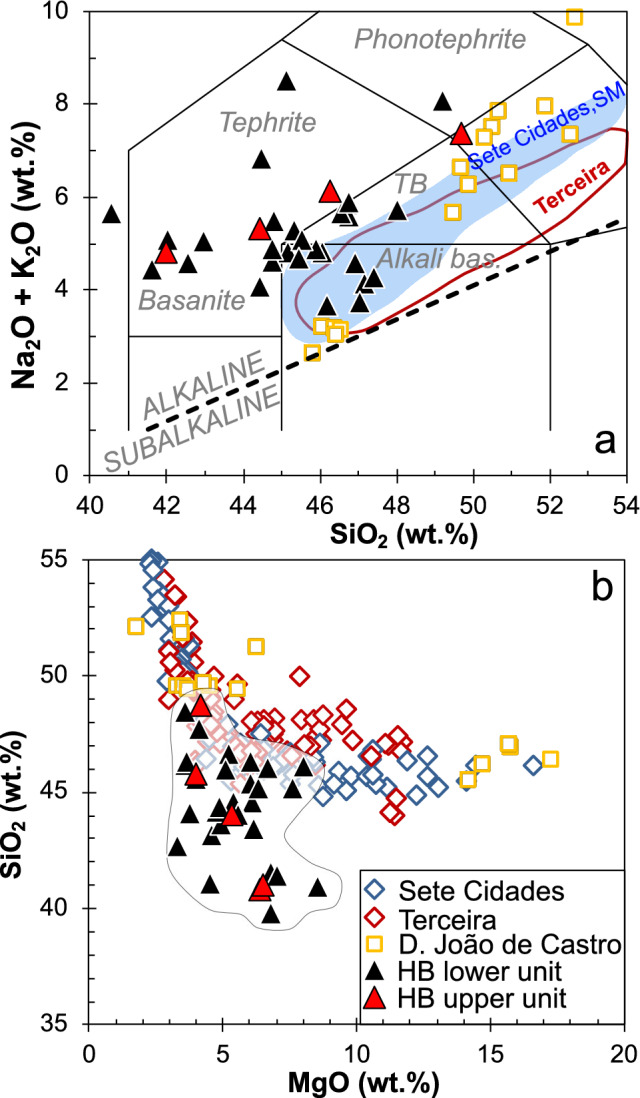
(**a**) Anhydrous total alkali contents versus SiO_2_ (TAS) classification after Le Maitre^55^ with subdivision in alkaline and subalkaline composition after MacDonald^56^ showing the lavas recovered from the flank of the Hirondelle Basin (HB) in comparison to those from Sete Cidades on São Miguel, Terceira, and D. João de Castro^17,57–62^; (**b**) Variation of SiO_2_ contents versus MgO showing relatively low SiO_2_ for a given MgO of the Hirondelle Basin flank lavas compared to lavas from the other young volcanoes of the Terceira Rift.

**Figure 4:**
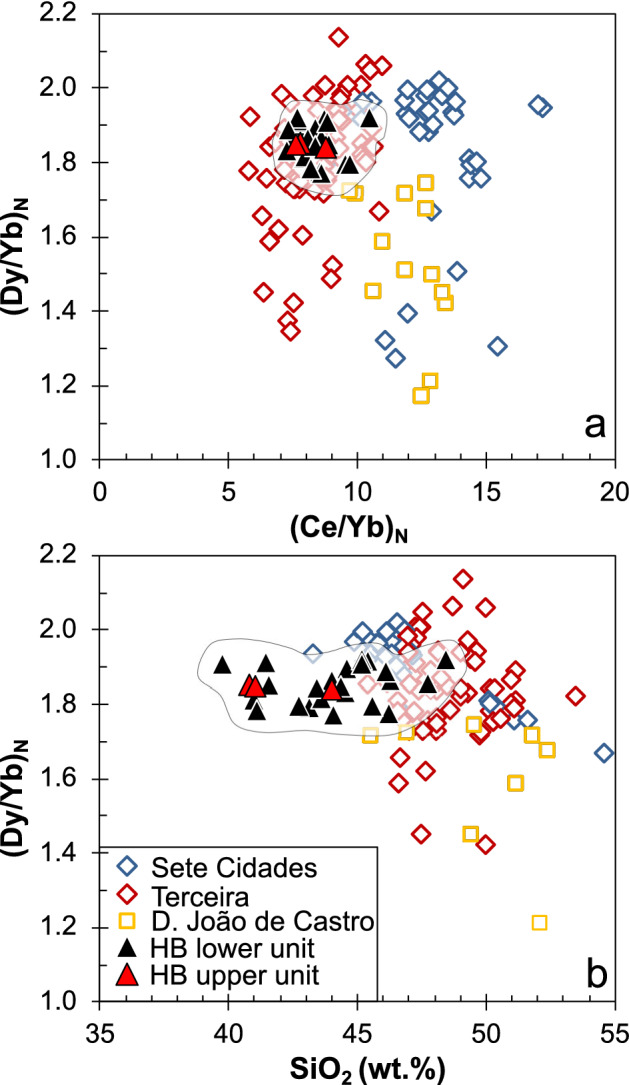
(**a**) Variation of the chondrite-normalized Dy/Yb versus Ce/Yb of the basalts from the Hirondelle Basin (HB) flank in comparison to those from the young volcanoes of the Terceira Rift; (**b**) Variation of (Dy/Yb)_N_ versus SiO_2_ contents of the older basalts from the HB flank to the young lavas. Note that the basalts from D. João de Castro seamount have higher SiO_2_ but lower (Dy/Yb)_N_ than the HB basalts. Data sources as in Fig. 3.

**Figure 5:**
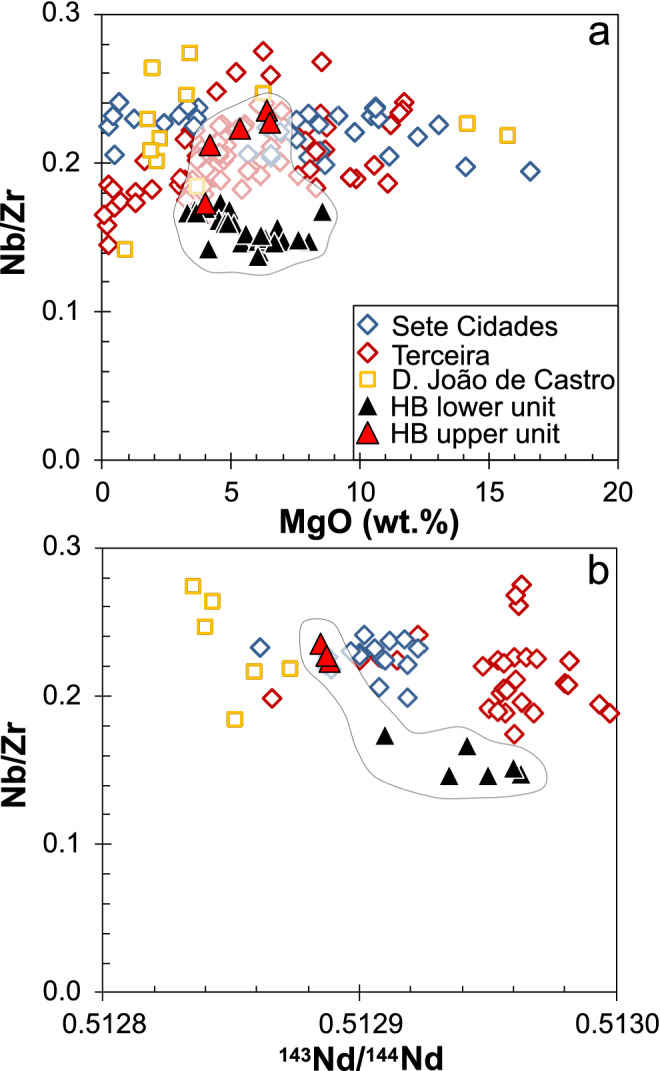
(**a**) Nb/Zr versus MgO and (**b**) Nb/Zr versus ^143^Nd/^144^Nd ratios for the lavas from the northern Hirondelle Basin (HB) compared to rocks from Terceira, Sete Cidades volcano on São Miguel, and D. João de Castro. Data sources as in Fig. 3.

**Figure 6:**
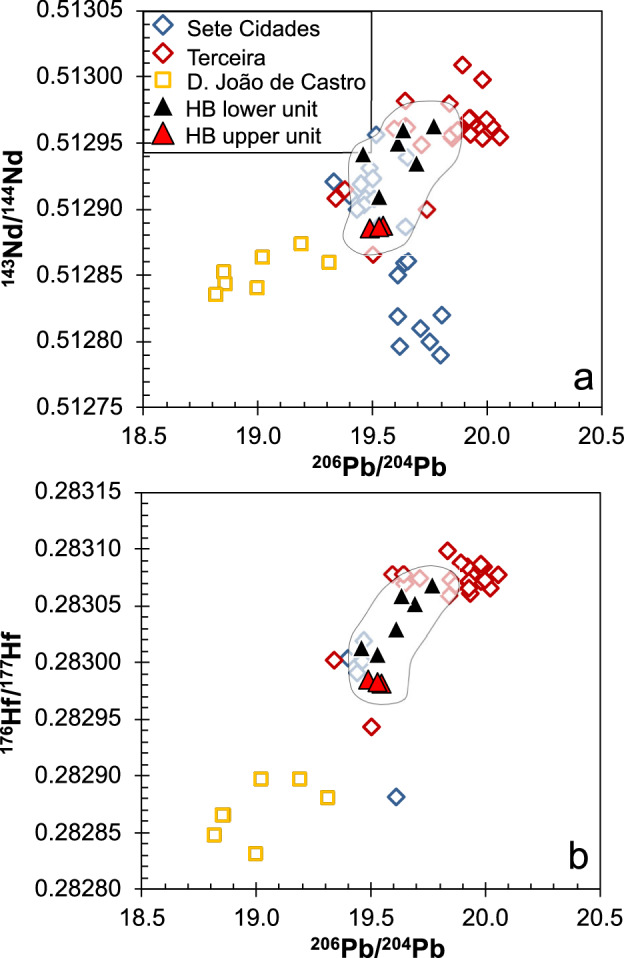
(**a**) ^143^Nd/^144^Nd versus ^206^Pb/^204^Pb and (**b**) ^176^Hf/^177^Hf versus ^206^Pb/^204^Pb ratios of the northern Hirondelle Basin (HB) lavas, compared to the data from Terceira, D. João de Castro, and Sete Cidades volcano on São Miguel. Data sources as in Fig. 3.

Major and trace element concentrations, as well as Sr–Nd–Hf and double spike Pb isotope ratios were analyzed at the GeoZentrum Nordbayern (see “Methods”). Two lava units are defined based on different Nb/Zr ratios: (1) lavas between 2510 and 1438 mbsl have Nb/Zr < 0.2, and (2) the uppermost four samples between 1390 and 1308 mbsl have Nb/Zr > 0.2 (Fig. 2a). Lavas with low Nb/Zr also display low TiO_2_ contents (< 4.2 wt% at > 4 wt% MgO) and relatively high ^176^Hf/^177^Hf isotope ratios (Fig. 2b). The lavas from the Hirondelle Basin wall are alkali basalts, basanites, tephrites, trachybasalts, and phonotephrites with 8.5 to 3.3 wt% MgO (Fig. 3a). Most of the lavas from the Hirondelle Basin wall have lower SiO_2_ contents at a given MgO concentration compared to lavas from the young volcanoes along the Terceira Rift (Fig. 3b). All lavas are enriched in light relative to heavy Rare Earth Elements (REE) with chondrite-normalized Ce/Yb ratios between 7 and 11 which is similar to basalts from Terceira, whereas lavas from Sete Cidades on São Miguel and from D. João de Castro seamount are more enriched (Fig. 4a). The basalts from the Hirondelle Basin have relatively high (Dy/Yb)_N_ that are comparable to Sete Cidades and Terceira lavas but the D. João de Castro alkali basalts have lower (Dy/Yb)_N_ and higher SiO_2_ than those from the Hirondelle Basin wall (Fig. 4b). The lavas of the upper unit between 1390 and 1274 mbsl with high Nb/Zr have low ^143^Nd/^144^Nd and ^176^Hf/^177^Hf ratios but high ^87^Sr/^86^Sr relative to lavas from the lower unit (Figs. 2 and 5). In terms of Nb/Zr and Nd isotope ratios the upper basalts resemble those from D. João de Castro and Sete Cidades whereas the lower lavas overlap with compositions of Terceira basalts (Fig. 5b). The ^206^Pb/^204^Pb ratios of the Hirondelle Basin lavas range from 19.46 to 19.77 where the lower unit generally has higher ratios than the upper unit (Fig. 6). The isotopic composition of the Hirondelle Basin flank lavas overlaps with those of rocks from Terceira but the low Nd and Hf isotope ratios of the upper unit basalts resemble Sete Cidades lavas. Samples from the young D. João de Castro seamount have even lower Nd, Hf, and Pb isotope ratios than the Hirondelle Basin flank basalts.

## Discussion

### Magmatic evolution of the Azores Plateau

Oceanic plateaus typically consist of tholeiitic lavas reflecting large degrees of melting in the shallow mantle^4,6^. The alkali basaltic to basanitic lavas forming the upper > 1 km of the crust at the Hirondelle Basin (Fig. 3a) are unlikely to represent the initial magmatic plateau-forming stage and differ significantly from > 5 Ma old tholeiitic lavas found on the western Azores Plateau^30^. Experimental results indicate that alkali basaltic to basanitic melts form by low degrees of partial melting (< 5%) of carbonated garnet peridotite at high pressures > 3 GPa^63,64^. The light REE enrichment supports low degrees of melting and the relative depletion of heavy REE in the Hirondelle Basin lavas (Fig. 4a) suggests a deep melting regime of the magmas in garnet peridotite stability field^65,66^. Thus, the alkaline composition of the lavas from the Hirondelle Basin crustal profile reflects deep partial melting beneath thick lithosphere, unlike the tholeiitic mid-ocean ridge basalts at ultraslow-spreading axes^67^. We conclude that the lavas from the Hirondelle Basin flank represent an alkaline magmatic phase suggesting formation of deep magmas beneath the lithosphere between ~ 2.0 and 1.5 Ma ago, rather than extensive shallow melting producing tholeiitic melts. The primitive basalts of the young D. João de Castro seamount have higher SiO_2_ contents and lower (Dy/Yb)_N_ than lavas of the Hirondelle Basin flank (Fig. 4b) which implies larger degrees of melting at lower pressures. Consequently, deep melting apparently formed the magmas prior to ~ 1.5 Ma, followed by lithospheric thinning due to tectonic rifting of the Hirondelle Basin, and finally the generation and eruption of the D. João de Castro magmas.

### Change of mantle sources with time

The Nb/Zr ratios are not affected by fractional crystallization processes because they remain constant over a large range of MgO contents (Fig. 5a). At similar MgO the upper unit lavas have higher Nb/Zr than the lower unit basalts (Fig. 5a). Additionally, Nd isotope ratios correlate with Nb/Zr implying that Nb/Zr variations reflect mantle source compositions (Fig. 5b). The lower Nb/Zr ratios of the Hirondelle Basin flank lavas compared to those of the islands indicate a more depleted source. Radiogenic isotope compositions suggest that volcanoes of the Azores are typically fed by distinct mantle sources^57,68^. The Hf and Nd isotope ratios are insensitive to alteration and thus imply different mantle sources between the two lava units of the Hirondelle Basin flank (Fig. 6). Most lavas from the lower unit have higher ^143^Nd/^144^Nd, ^208^Pb/^204^Pb, and ^206^Pb/^204^Pb compositions than those from the upper unit. The isotopes indicate a transition from a source resembling that of Terceira^57,58^ towards one with lower Nd and Pb isotope ratios (Fig. 6), possibly reflecting a source similar to Sete Cidades on western São Miguel or D. João de Castro magmas. The change in isotope composition confirms the large variation and small scale of the isotopic mantle heterogeneity in the Azores^57,68^. We conclude that the two lava units recovered from the northern Hirondelle Basin rift flank show that the mantle beneath the Hirondelle Basin changed from a source comparable to that of Terceira magmas towards one closer to recent Sete Cidades and/or D. João de Castro seamount volcanism, implying rapid replacement of heterogeneous mantle in the melting zone of the Terceira Rift. Comparable changes of the mantle sources are observed at other volcanoes in the Azores, for example, at São Jorge^17^. The change in composition of the Hirondelle Basin magmas apparently coincides with less frequent lava eruptions in the upper part of the profile. This suggests a decrease of magmatic activity and possibly decreasing melt production in the mantle at ~ 1.6 Ma prior to rifting of the Hirondelle volcanic structure. Volcanic eruptions and thus possibly also magma formation then recommenced in the Hirondelle Basin at D. João de Castro seamount after the rift basin had formed.

### Constraints on the extension process of the Terceira Rift

No systematic magnetic patterns were observed along the Terceira Rift but the magnetic anomalies are different to the strike of the MAR and parallel to the young volcanic structures of Graciosa, Terceira, D. João de Castro, and Sete Cidades^35^. These anomalies were interpreted as indication of spreading of 160 to 75 km of new crust either since chron 13 (~ 36 Ma) or chron 6 (~ 20 Ma)^13,35^. However, seismic profiles across the southeastern Terceira Rift show faulted crust but no evidence for young magmatic spreading^14^ which is in agreement with structural observations on São Miguel island^15^. A teleseismic receiver function study reveals that the lithosphere beneath Terceira and São Miguel islands has a thickness of ~ 80 km implying rifting did not cause significant thinning of the plate^27^. Additionally, the alkaline basaltic composition of the lavas erupting at the Hirondelle Basin in the past 2 million years implies melting beneath thick lithosphere, i.e. there is no geochemical evidence for lithospheric thinning with production of tholeiitic magmas and formation of new magmatic crust and underlying lithospheric mantle. Rather, the uppermost ~ 1.2 km thick alkaline lava pile of the Hirondelle Basin flank erupted on top of existing thick lithosphere within 450 kyrs which is comparable to the estimated 250–600 kyrs for formation of the volcanic layer 2A at slow-spreading mid-ocean ridges^69^. Ultraslow-spreading axes show alternating amagmatic extensional phases and magmatic phases with extension by dike intrusion^23^. Although we do not find evidence for the formation of new lithosphere by magmatic processes in the Hirondelle Basin, we agree with Sibrant et al.^15^ that the extension of Terceira Rift follows patterns similar to other ultraslow mid-ocean ridges^70^.

**Figure 7:**
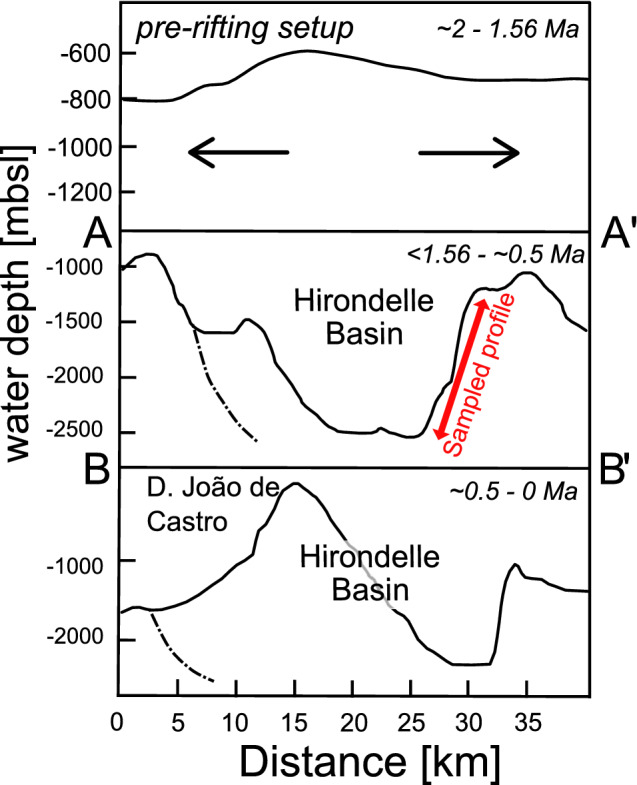
Cross section (SW–NE) of the distinct formation phases from top to bottom. The uppermost sketch shows the assumed first phase with the pre-rifting volcanic construction. The second diagram shows the opening of the basin through tectonic processes along profile A–A’ in Fig. 1. The location of the sampled profile is shown at the north-eastern graben shoulder. The lowermost diagram shows the present situation along profile B–B’ in Fig. 1. The new volcanic construction phase since perhaps 0.5 Ma formed the submarine seamount D. João de Castro.

The crust exposed at the Hirondelle Basin may thus represent the early magmatic phase in the building of a volcanic ridge (Fig. 7). This volcanic ridge was split by tectonic rifting younger than 1.56 Ma that formed the Hirondelle Basin (Figs. 1 and 7 cross section: A–A’) and at a time when the Terceira Rift in this region became volcanically inactive. More recently, the formation of volcanic edifices like D. João de Castro seamount along the Terceira Rift (Figs. 1 and 7 cross section: B–B’) indicates that magmas are focusing beneath this portion of the rift leading to volcanism and lateral dike intrusion, potentially with some magmatic spreading in the shallow crust. Our new age of < 1.56 Ma for the opening of Hirondelle Basin is in agreement with previous estimates of the onset of Terceira Rift extension between 1.8 and 0.8 Ma further to the west^16^, and between 2.7 and 1.4 Ma further to the east^15^. The onset of volcanic activity in the Hirondelle Basin is unknown and we assume that D. João de Castro seamount formed within the past 500 kyrs similar to the youngest volcanoes on Terceira and São Miguel^17,19,71^. Rifting of volcanic structures followed by formation of young volcanic cones has also been observed at the eastern end of Terceira^36^ and on several other islands with the Terceira Rift like on Graciosa^72^. Similar episodic magmatic phases along an ultraslow-spreading axis exist at the Southwest Indian Ridge^23^. We conclude that the lavas from the northern rift shoulder of the Hirondelle Basin neither represent formation of new ocean floor by magmatic spreading as previously suggested^13,35^, nor do the samples represent an initial phase of formation of the Azores Plateau by high degrees of melting in a mantle plume. Rather, the lavas of the uppermost crust exposed along the Hirondelle Basin represent a rifted volcanic structure that formed by episodic deep and low degrees of partial melting. The volcanic succession implies that much of the thickening (> 1 km) of the eastern Azores Plateau occurred by late addition of lavas. Dike intrusions into the crust and potential magmatic spreading are probably restricted to the volcanic centers of the Azores islands^16^ and D. João de Castro seamount. We speculate that the wide zone of extension observed in the Azores Plateau^12,17^ may become focused along the narrow Terceira Rift with four magmatic segments at western São Miguel, D. João de Castro seamount, Terceira, and Graciosa (Fig. 1). The magma intrusions weaken the oceanic lithosphere which in turn causes strain localization^24,25^. Thus, the general pattern of extension of the Azores Plateau resembles that of continental rifts where tectonic extension starts in a relatively wide area along boundary faults with later narrowing of the zone of deformation and active volcanism^73^.

## Conclusions

The upper ~ 1.2 km of the Azores Plateau crust along the Hirondelle Basin formed within ~ 500 kyrs with the lower 1000 m-thick portion erupting within 350 kyrs. Thus, magmatic eruption volumes decreased significantly to the top while the magma source compositions changed. The Hirondelle Basin shows similar episodic volcanic phases to ultraslow-spreading axes although the lithosphere is much thicker and the alkaline basaltic magmas suggest deep melting at relatively low degrees. The formation of volcanoes with heights of > 1 km is followed by tectonic extension with normal faulting but there is no evidence for magmatic spreading with production of new basaltic crust. Slight changes in basalt composition from mainly basanites prior to 1.56 Ma to recent alkali basalts at the D. João de Castro seamount may indicate increasing degrees of melting due to thinning of the lithosphere associated with the formation of the Terceira Rift. The episodic volcanism along the Terceira Rift with breaks of perhaps 1 million years reflects variations of magma formation in the mantle possibly reflecting the ascent of fertile mantle into the melting zone. The tectonic and magmatic evolution of the Hirondelle Basin of the Terceira Rift thus resembles that known from narrow continental rift systems^24^.

## Supplementary information

Supplementary Material I.

Supplementary Material II.

Supplementary Material III.

Supplementary Material IV.

## Data Availability

The dataset we used in the study can be found in Supplementary Information of the manuscript.
