# Supplementary material for: Rifting of the oceanic Azores Plateau with episodic volcanic activity
Source: Sci Rep. 2020 Nov 12;10:19718. doi: 10.1038/s41598-020-76691-1 (PMC7665008; doi:10.1038/s41598-020-76691-1)
Supplement: Supplementary file 1 — Supplementary Material I. [file 41598_2020_76691_MOESM1_ESM.pdf]

**RIFTING OF THE OCEANIC AZORES PLATEAU WITH EPISODIC VOLCANIC ACTIVITY**

B. Storch*[1], K.M. Haase[1], R.H.W. Romer[1], C. Beier[1,2], A.A.P. Koppers[3]

[1]GeoZentrum Nordbayern, Friedrich-Alexander-Universität Erlangen-Nürnberg (FAU) Schlossgarten 5, 91054 Erlangen, Germany *(corresponding author: Bettina.storch@fau.de)*

[2]Department of Geosciences and Geography, Research programme of Geology and Geophysics (GeoHel), PO Box 64, FIN-00014 University of Helsinki, Finland

[3]College of Earth, Ocean and Atmospheric Sciences, Oregon State University, 104 CEOAS Admin Bldg, Corvallis, OR 97331-5503 USA

**SUPPLEMENTARY MATERIAL I**

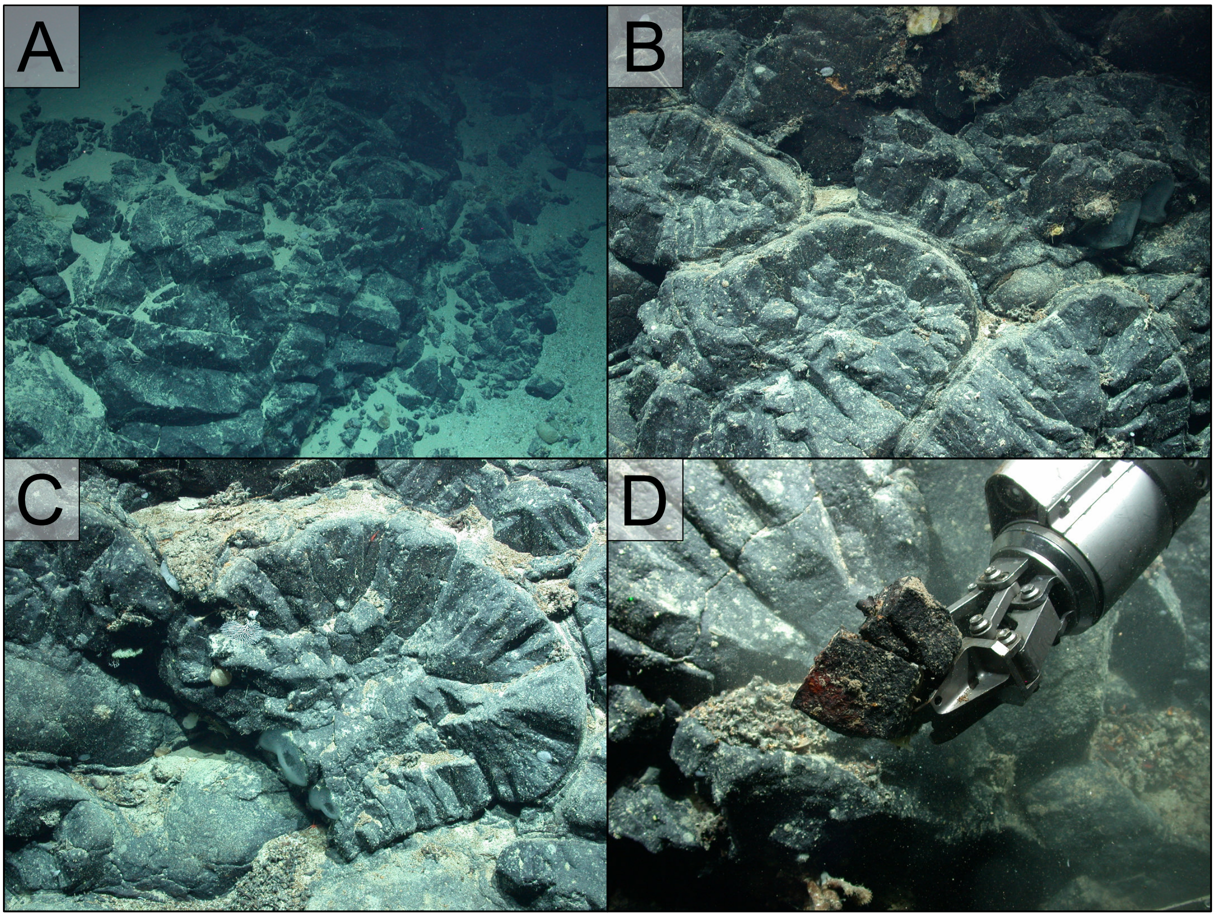


*Supplementary Figure I:* photographic images of dive 399 during cruise M128 at the northern graben shoulder in the Hirondelle Basin, showing fresh outcropping lava flows (A) and pillow basalts (B,C,D). Photos and Samples were taken with the ROV "QUEST 4000" from MARUM *(photo credits and copyright: "MARUM - Center for Marine Environmental Sciences, University of Bremen").*
