# Supplementary material for: Rifting of the oceanic Azores Plateau with episodic volcanic activity
Source: Sci Rep. 2020 Nov 12;10:19718. doi: 10.1038/s41598-020-76691-1 (PMC7665008; doi:10.1038/s41598-020-76691-1)
Supplement: Supplementary file 2 — Supplementary Material II. [file 41598_2020_76691_MOESM2_ESM.pdf]

RIFTING OF THE OCEANIC AZORES PLATEAU WITH EPISODIC VOLCANIC ACTIVITY

B. Storch*[1], K.M. Haase[1], R.H.W. Romer[1], C. Beier[1,2], A.A.P. Koppers[3]

[1]GeoZentrum Nordbayern, Friedrich-Alexander-Universität Erlangen-Nürnberg (FAU) Schlossgarten 5, 91054 Erlangen, Germany *(corresponding author: Bettina.storch@fau.de)*

[2]Department of Geosciences and Geography, Research programme of Geology and Geophysics (GeoHel), PO Box 64, FIN-00014 University of Helsinki, Finland

[3]College of Earth, Ocean and Atmospheric Sciences, Oregon State University, 104 CEOAS Admin Bldg, Corvallis, OR 97331-5503 USA

SUPPLEMENTARY MATERIAL II

Methods

Major elements

Most of the samples from ROV Dives 390 and 399 (collected with MARUM Quest 4000) are plagioclase ± clinopyroxene phyric, and only few aphyric, moderately to highly vesicular volcanic rocks. Only one glass sample could be separated (IEAZO1047; 789ROV-01) and was prepared for microprobe analysis using the JEOL JXA-8200 SuperProbe Electron Probe Microanalyzer (EPMA) at the GeoZentrum Nordbayern in Erlangen, Germany method after [1]. Weathered surfaces and vesicle fillings were removed from the whole-rock samples prior to sample preparation. The samples were then washed, coarse crushed and powdered in an agate grinder. We carried out major and trace elements, as well as Sr, Nd, Hf and high-precision Pb (double spike) isotope ratios as well at the Bioforschungszentrum, in the laboratories of the GeoZentrum Nordbayern, in Erlangen, Germany. For the whole-rock major element analysis 1.00 g rock powder (dried at 105° C) were mixed with 4.83 g lithium tetraborate ($Li_2B_4O_7$) and 230 mg di-iodpentoxide (I_2O_5) then fused to a homogenous glass tablet and measured with SPECTRO XEPOS HE x-ray spectrometer (Spectro Analytical Instruments GmbH). International rock standards BHVO-2, BE-N, BR and GA were analyzed with the samples (suppl. III & IV).

Trace elements

The trace elements were measured with inductively coupled plasma mass spectrometry (ICP-MS) using a Thermo Scientific XSERIES 2 Quadrupole ICP-MS at the GeoZentrum Nordbayern in Erlangen. For the trace element analysis ~0.05 g of whole rock sample powder was accurately weighed into SAVILLEX PFA vials, wetted with a few drops 15M HNO_3, before adding 3 ml 12M HF. The sealed beaker sat on a hotplate (<100°C) for 24h. After adding 6 drops concentrated perchloric acid, the solution was evaporated, then 2 ml 15M HNO3 was added and fumed off (120°C). This step was repeated twice with increasing temperature (140-

155°C). The dried residue was redissolved in 4ml 15M HNO_3, 2 drops 12M HF and 4 ml MQ H_2O and sat on a hotplate for 12h. The fully dissolved sample solution was further diluted with MQ H_2O to a final total weight of 200 g, to reach a dilution factor of 4,000. The final solution was mixed with an internal standard solution (containing Be-In-Rh-Bi), and introduced into the plasma via a CETAC Aridus II Desolvating Nebulizer System. A mixed Li-Ce-In-U solution was used to tune the instrument to obtain a maximum sensitivity (typically 400,00 cps/ppb for ^{238}U) and low oxide production (Ce/CeO>5,000). The Hawaiian basalt standard (BHVO-2, US Geological Survey) were processed and measured with the samples (trace element and isotope ratios) to determine the accuracy and reproducibility of our measurements (see supplementary data table III).

Isotopes

For isotope ratio analysis, about 0.10 to 0.12 g dried whole rock sample powder was leached with 6M HCl for 1h, then rinsed twice with MQ H_2O. The glass sample was leached with 2.5M HCl, H_2O_2 and MQ H_2O for 10 min and also rinsed twice with MQ H_2O. A15M HNO_3 and 12M HF solution was added to the sample and placed onto hotplate overnight, to prepare the samples for column procedure. The solution was evaporated and redissolved 6M HCl, dried down and finally dissolved in double-distilled 1M HCl. The solution was centrifuged and only the clear solution was transferred onto 100 μl Eichrom Sr Resin filled columns. Each sample ran through the columns twice. The final Pb-fraction was dried down and redissolved in 2 ml 2% HNO3. Lead isotope measurements were carried out on a Thermo Scientific Neptune Plus High Resolution Multicollector ICP-MS (MC-ICP-MS) at the Bioforschungszentrum, in the laboratories of GeoZentrum Nordbayern, in Erlangen, Germany. A ^{207}Pb – ^{204}Pb double spike was used to correct for instrumental mass fractionation. The double spike, with a $^{207}Pb/^{204}Pb$ ratio of 0.8135, was calibrated against a solution of the NBS982 equal atom Pb standard. Spiked and unspiked sample solutions were introduced into the plasma via a CETAC Aridus Desolvating Nebulizer System, and measured in static mode. Interference of ^{204}Hg on mass 204 was corrected by monitoring ^{202}Hg. An exponential mass fractionation correction was applied offline using the iterative method of Compston and Oversby [2], the correction was typically 4.5 permil per amu. The measurements of the NBS981 Pb isotope standard gave $^{206}Pb/^{204}Pb$, $^{207}Pb/^{204}Pb$, $^{208}Pb/^{204}Pb$ ratios of 16.9391 ± 0.0018, 15.4965 ± 0.0019 and 36.7149 ± 0.0036.

The wash from the Pb columns (containing amongst others Hf-Sr-Nd) was dried and redissolved in 3.5M HNO_3. Columns filled with 100 μl Eichrom Sr Resin and 150 μl Eichrom TRU Resin were prepared, washed and the columns with TRU Resin were positioned above the Sr Resin columns. The samples solution was loaded onto the upper columns and dripped directly onto the lower Sr Resin filled columns. The resin was washed and the racks decoupled before collecting Sr and Nd fractions separately. The Nd fraction was dried and redissolved in 0.5 ml 0.25M HCl, then passed through columns filled with 1.5 ml Eichrom LN Resin to separate Nd from other REE. The Sr fractions were dried and loaded onto tantalum single filaments with 1M H_3PO_4. The collected Nd cuts and the 'Erlangen Nd' standard were loaded onto a Ta filament (double filament carrier) with 0.1M H_3PO_4, facing a Re filament. Strontium and Nd isotopes were measured using a Thermo Scientific Triton Series Multicollector Thermal Ionization Mass Spectrometer (TIMS) in static mode at the Bioforschungszentrum, in the laboratories of GeoZentrum Nordbayern, in Erlangen. Strontium isotope measurements were corrected for instrumental mass fractionation assuming $^{88}Sr/^{86}Sr$ = 0.1194, and corrected for the

contribution of ^{87}Rb to mass 87. Neodymium isotope data were corrected for mass fractionation using a ^{146}Nd/^{144}Nd ratio of 0.7219. Samarium interference on mass 144 was corrected by measuring ^{147}Sm. During the course of this study the NBS987 Sr standard yielded 0.710259, the 'Erlangen Nd' standard yielded 0.511840, equivalent to a value of 0.511850 for the La Jolla standard.

Hafnium was isolated using a modified version of the methods from Bast, et al. [3] and Münker, et al. [4]. The Pb-washes containing Hf were dried and redissolved in 1M HCl + 0.1M HF, then passed through cation columns. The Hf-fraction was dried down overnight with H_2O_2 at 80°C, then diluted again with an oxidation mixture and loaded onto Eichrom LN Resin (1.5 ml) filled columns. In further steps Ti was washed out, as well as H_2O_2, to prevent the formation of bubbles in the resin. Before collecting Hf with 6M HCl-0.2M HF, Zr was separated (using 6M HCl-0.06M HF). The method is described in detail in Storch et al. (in prep.). The isotopes were measured using a Thermo Scientific Neptune Plus High Resolution Multicollector ICP-MS in static mode. Interfering isotopes of Lu and Yb were monitored and corrected during the measurement, but were negligible. The presented data are reported relative to a ^{176}Hf/^{177}Hf value of 0.282169 for the AMES Grenoble Hf standard [5].

<u>^{40}Ar/^{39}Ar ages</u>

All ^{40}Ar/^{39}Ar age determinations (groundmass and plagioclase phenocrysts) for the Hirondelle Basin samples were carried out at the Oregon State University (OSU) Argon Geochronology Laboratory, USA. The processing of the age data was similar to the method in Reagan, et al. [6]. The whole rock samples (weathered surfaces and vesicle fillings removed in advance at the GeoZentrum Erlangen) were crushed using a steel plated jaw crusher and sieved. The separated grain size fraction between 150-300 μm was then washed (pure water), dried at 55°C and plagioclase phenocrysts separated through hand-picking from groundmass material. The density fractions were acid-leached with 1M HCl, then 6M HCl, 1M HNO_3, 3M HNO_3 and rinsed with ultra-pure deionized water (all for about 60 min) in an ultrasonic bath heated to ~ 50°C. The plagioclase phenocrysts were further leached using 5% HF for 5-15 min. The leached samples were irradiated for 6 h in the TRIGA nuclear reactor at OSU, together with the FCT sanidine flux monitor [7]. The individual J-values for each sample were calculated by parabolic extrapolation of the measured flux gradient against irradiation height and typically give 0.1-0.2% uncertainty (1σ). The ^{40}Ar/^{39}Ar incremental heating age was determined with two multi-collector ARGUS-VI mass spectrometers. The ARGUS-VI-D is equipped with 5 Faraday collectors (all fitted with 10^{12} Ω resistors) and 1 ion-counting CuBe electron multiplier, which is located next to the lowest mass Faraday collector. The ARGUS-VI-E has 5 Faraday collectors (two fitted with 10^{12} Ω resistors for mass 41, 40 and three with 10^{13} Ω resistors for mass 39, 38, 37) and also 1 ion-counting CuBe electron multiplier, again next to the lowest mass Faraday collector. Due to this setup a simultaneous measurement of all argon isotopes, with mass 36 on the multiplier and masses 37 trough 41 on the five adjacent Faradays is possible. After loading the irradiated samples into Cu-planchettes in an ultra-high vacuum sample chamber, they were incrementally heated by scanning a defocused 25 W CO_2 laser beam in preset patterns across the sample, in order to release the argon evenly. Each pass involved incremental heating of 15-20 mg of separated groundmass material or plagioclase phenocrysts. The sample material was 'pre-cleaned' then for 60 s, while released gasses were pumped away directly, at two low (0.5%,

1.8%) laser power settings to remove any loosely-held atmospheric Argon adsorbed onto grain surfaces. After heating, the reactive gases were cleaned out using a SAES Zr-al ST101 getter operated at 400°C and two SAES Fe-V-Zr ST172 getters operated at 200°C and room temperature, respectively. Samples were held in the extraction line for a total time of 6 min. Blank intensities were measured every 3 incremental heating steps for groundmass and glass, and every 2 steps for plagioclase phenocrysts. For calculating the ages, the corrected decay constant of Steiger and Jäger [8]was used: 5.530 ±0.097 x 10^{-10} yr^{-1} (2σ) as reported by Min, et al. [9]. Incremental heating plateau ages and isochron ages were calculated as weighted means with $1/\sigma^2$ as weighting factor [10] and as YORK2 least-square fits with correlated errors [11] using the ArArCALC v2.7.0 software from Koppers [12] available from the http://earthref.org/ArArCALC/ website. The samples were initially interpreted using the inverse isochron because such ages do not assume a $^{40}Ar/^{36}Ar$ composition for trapped Ar. Inverse isochron ages are calculated for samples with five or more data points, using steps that deviate by no more than 3σ from the $^{39}Ar/^{40}Ar$ and $^{36}Ar/^{40}Ar$ weighted means with a uniform distribution [13].In addition, the isochron ages are considered robust if (1) the total released ^{39}Ar (k) ≥ 50%. (2) the isochron has a spreading factor > 5% S-factor; [13], MSWD <1 + 2 $(2/f)^{1/2}$ [14], where f=n-2 and n is the number of steps in the isochron, and (3) the $^{40}Ar/^{36}Ar$ intercept is within error or greater than 295.5 ±0.7 1σ. If experiments had no resolvable isochron but yielded highly radiogenic argon, the initial trapped $^{40}Ar/^{36}Ar$ was assumed to equal 295.5 [15], and a plateau model age was calculated.

The groundmass ages are interpreted as eruption ages (supplementary material III: data table & IV: age data presentation), yet from the inverse isochron $^{40}Ar/^{36}Ar$ intercept calculations, the samples do show evidence for (minor amounts of) excess argon, which has been corrected accordingly cf. [16]. After correction for this trapped Argon signature, the plateaus are between 56-100% wide, showing low MSWD values between 0.34-1.85. These groundmass ages are high precision with typical 2σ uncertainties of only 5-13 ka (~3-6 ‰). Two samples also have dates for the phenocrystic plagioclase phase, with mixed success. Sample IEAZO1054 has a concordant (but 10x less precise) plagioclase plateau age of 1.877.3 ± 0.091 Ma, whereas sample IEAZO1064 has a much older plagioclase plateau age of 1.979 ± 0.051 Ma compared to the more precise 1.636 ± 0.005 Ma groundmass age. In this latter case, the stacked inverse isochron data shows clear evidence for the plagioclase being contaminated with excess argon, increasing its overall apparent age. In this study we thus focus on the resulting eruption ages from the groundmass analyses.

Reference List

1 Brandl, P. A. *et al.* Volcanism on the flanks of the East Pacific Rise: Quantitative constraints on mantle heterogeneity and melting processes. *Chemical Geology* **298**, 41-56, doi:10.1016/j.chemgeo.2011.12.015 (2012).

2 Compston, W. & Oversby, V. M. Lead isotopic analysis using a double spike. *Journal of Geophysical Research* **74**, 4338-4348, doi:10.1029/JB074i017p04338 (1969).

3 Bast, R. *et al.* A rapid and efficient ion-exchange chromatography for Lu-Hf, Sm-Nd, and Rb-Sr geochronology and the routine isotope analysis of sub-ng amounts of Hf by MC-ICP-MS. *The Royal Society of Chemistry* **30**, 2323-2333 (2015).

4 Münker, C., Weyer, S., Scherer, E. E. & Mezger, K. Separation of high field strength elements (Nb, Ta, Zr, Hf) and Lu from rock samples for MC-ICPMS measurements. *Geochemistry Geophysics Geosystems* **2** (2001).

5 Chauvel, C., Bureau, S. & Poggi, C. Comprehensive Chemical and Isotopic Analyses of Basalt and Sediment Refernce Materials. *Geostandards and Geoanalytical Research* **35**, 125-143 (2011).

6 Reagan, M. K. *et al.* Forearc ages reveal extensive short-lived and rapid seafloor spreading following subduction

initiation. *Earth and Planetary Science Letters* **506**, 520-529, doi:10.1016/j.epsl.2018.11.020 (2019).
7 Kuiper, K. F. *et al.* Synchronizing rock clocks of Earth history. *Science* **320**, 500-504, doi:10.1126/science.1154339 (2008).
8 Steiger, R. H. & Jäger, E. Subcommission on geochronology: Convention on the use of decay constants in geo- and cosmochronology. *Earth and Planetary Science Letters* **36**, 359-362, doi:10.1016/0012-821x(77)90060-7 (1977).
9 Min, K., Mundil, R., Renne, P. R. & Ludwig, K. R. A test for systematic errors in 40Ar/39Ar geochronology through comparison with U/Pb analysis of a 1.1-Ga rhyolite. *Geochimica et Cosmochimica Acta* **64**, 73-98, doi:10.1016/s0016-7037(99)00204-5 (2000).
10 Taylor, J. R. *An Introduction to Error Analysis: The Study of Uncertainties in Physical Measurements*. 2nd ed edn, (University Science Books, 1997).
11 York, D. Least squares fitting of a straight line with correlated errors. *Earth and Planetary Science Letters* **5**, 320-324, doi:10.1016/s0012-821x(68)80059-7 (1968).
12 Koppers, A. A. P. ArArCALC—software for 40Ar/39Ar age calculations. *Computers & Geosciences* **28**, 605-619, doi:10.1016/s0098-3004(01)00095-4 (2002).
13 Jourdan, F., Renne, P. R. & Reimold, W. U. An appraisal of the ages of terrestrial impact structures. *Earth and Planetary Science Letters* **286**, 1-13, doi:10.1016/j.epsl.2009.07.009 (2009).
14 Wendt, I. & Carl, C. The statistical distribution of the mean squared weighted deviation. *Chemical Geology: Isotope Geoscience section* **86**, 275-285, doi:10.1016/0168-9622(91)90010-t (1991).
15 Nier, A. O. A Redetermination of the Relative Abundances of the Isotopes of Carbon, Nitrogen, Oxygen, Argon, and Potassium. *Physical Review* **77**, 789-793, doi:10.1103/PhysRev.77.789 (1950).
16 Heaton, D. E. & Koppers, A. A. P. High-resolution 40Ar/39Ar geochronology of the Louisville Seamounts IODP Expedition 330 drill sites: Implications for the duration of hotspot-related volcanism and age progressions. *Geochemistry, Geophysics, Geosystems*, doi:10.1029/2018GC007759 (2019).
